# Heterotrophic Selenium Incorporation into *Chlorella vulgaris* K-01: Selenium Tolerance, Assimilation, and Removal through Microalgal Cells

**DOI:** 10.3390/foods13030405

**Published:** 2024-01-26

**Authors:** Zhenyu Zhang, Yan Zhang, Yanying Hua, Guancheng Chen, Pengcheng Fu, Jing Liu

**Affiliations:** 1 State Key Laboratory of Marine Resource Utilization in South China Sea, Hainan University, Haikou 570228, China; 2 International School of Public Health and One Health, Hainan Medical University, Haikou 571199, China

**Keywords:** *Chlorella*, organic selenium, heterotrophic cultivation, microalgae, biomass

## Abstract

*Chlorella* has been applied in the production of selenium (Se) enriched organic biomass. However, limited information exists regarding heterotrophic selenium tolerance and its incorporation into *Chlorella*. This study aimed to investigate the potential of using *Chlorella vulgaris* K-01 for selenium biotransformation. To assess the dose-response effect of Se stress on the strain, time-series growth curves were recorded, growth productivity parameters were calculated, and Gaussian process (GP) regression analysis was performed. The strain’s carbon and energy metabolism were evaluated by measuring residual glucose in the medium. Characterization of different forms of intracellular Se and residual Se in the medium was conducted using inductively coupled plasma-mass spectrometry (ICP-MS) and inductively coupled plasma optical emission spectrometer (ICP-OES). The EC50 value for the strain in response to Se stress was 38.08 mg/L. The maximum biomass productivity was 0.26 g/L/d. GP regression analysis revealed that low-level Se treatment could increase the biomass accumulation and the carrying capacity of *Chlorella vulgaris* K-01 in a heterotrophic culture. The maximum organic Se in biomass was 154.00 μg/g DW. These findings lay the groundwork for understanding heterotrophic microalgal production of Se-containing nutraceuticals, offering valuable insights into Se tolerance, growth dynamics, and metabolic responses in *Chlorella vulgaris* K-01.

## 1. Introduction

Selenium (Se) is an essential mineral nutrient and a double-edged sword in terms of its effects on the health of organisms [1,2,3,4]. While a daily intake of 40 μg is beneficial for adults, a 10-fold increase in the intake can lead to severe adverse effects [5], such as selenosis with stagnation in the respiratory and cardiovascular system and potential fatality [6]. Therefore, supplementation of inorganic Se should be conducted carefully as it could easily reach a toxic concentration [7]. Organic Se has been reported to have better bioavailability and be less toxic than inorganic Se forms [8], and it has an important relationship with thyroid metabolism, antioxidative capacity, and redox signaling [9]. Supplementation of Se could prevent Kashin–Beck disease, severe cardiomyopathy (Keshan disease), muscle disorders, and myxoedematous cretinism and mitigate type 2 diabetes, hepatopathies, and male infertility in humans [10,11,12].

Microalgae are rich in protein, making them capable of producing the organic Se in the form of selenoproteins, which is conducive to humans and animals, and it is a promising resource to develop nutraceuticals [13,14,15,16]. *Chlorella* spp., rich in essential amino acids, fatty acids, proteins, and vitamins [17], is listed on the Generally Recognized as Safe (GRAS) inventory, a regulatory framework for substances intended for use in human food or animal food, established by the FDA (https://www.fda.gov/food/gras-notice-inventory/recently-published-gras-notices-and-fda-letters accessed on 5 December 2023).

*Chlorella* spp. biomass has been utilized in industrial applications to produce functional beverages, yogurt, and cookies [18,19,20,21]. In addition, *Chlorella* has been extensively studied for its potential in developing value-added products such as polypeptides and polysaccharides [22,23]. Its supplementation has been found to exhibit various pharmacological activities in humans, including hypolipidemic, antitumor, antidiabetic, antihypertensive, and anti-asthmatic effects [23,24]. *Chlorella* spp. possesses the ability to convert inorganic Se into organic Se [25], with a reported Se bioavailability of around 50–60% [26]. The organic Se in *Chlorella* mainly consist of selenoprotein and selenium-containing polysaccharides, which showed enhanced antitumor, immune-enhancement, and antioxidant activities [27,28,29]. Therefore, *Chlorella* spp. is a promising starting point to develop Se-enriched nutraceuticals [30,31,32].

Previous studies have primarily focused on the microalgal enrichment of Se under photoautotrophic conditions, where light is used as an energy source and CO_2_ as a carbon source. However, there is limited research available on heterotrophic *Chlorella* cultivation for Se incorporation. Heterotrophic cultivation, utilizing glucose as both the carbon and energy source, without reliance on light, presents an alternative way for the cultivation of microalgae in regions with unfavorable climatic conditions and insufficient sunlight. It has the advantages of fast cell growth rate and high cell density [33]. Heterotrophic growth of *Chlorella* requires smaller facilities and offers approximately 5.5 times higher biomass yields compared to photoautotrophic conditions. Additionally, the scale-up period for heterotrophic microalgae is significantly shorter [34]. Moreover, heterotrophic cultures of *Chlorella* with Se have the potential to produce microalgal biomass with a higher content of organically bound Se [35].

In a previous study, *Chlorella vulgaris* K-01, identified from a brine graduation tower efflux, exhibited resistance to various abiotic stresses [36]. Given the limited understanding of Se stress responses and its incorporation into microalgal cell incorporation under heterotrophic conditions, this study aims to investigate the growth response of *Chlorella vulgaris* K-01 under various Se concentrations. Time-series growth curves were recorded to evaluate growth productivity, and Gaussian process regression analysis was performed. The carbon and energy metabolism of the strain were monitored by measuring residual glucose in the medium. Different forms of intracellular Se and residual Se in the medium were characterized using inductively coupled plasma-mass spectrometry (ICP-MS) and inductively coupled plasma optical emission spectrometer (ICP-OES). The findings from this study lay a foundation for developing a strategy for heterotrophic Se-containing microalgal biomass production, offering potential applications in nutraceutical development.

## 2. Materials and Methods

### 2.1. Heterotrophic Culture and Selenium Treatment

Heterotrophic medium (KC-H) was formulated per liter with 5 g of glucose, 0.405 g of KNO_3_, 0.235 g of NaCl, 0.235 g of NaH_2_PO_4_ 2H_2_O, 0.18 g of Na_2_HPO_4_·12H_2_O, 0.125 g of MgSO_4_·7H_2_O, 0.003 g of FeSO_4_·7H_2_O, 0.01 g of CaCl_2_·7H_2_O, and 1 mL of trace elements solution following Rippka’s protocol [37]. Distilled ultra-pure water was used for dilution, and the final pH of the medium was adjusted to 6.4 before autoclaving at 121 °C for 20 min for sterilization [31]. A 0.438 g selenite stock solution (1000 mg/L Se) was prepared and filtered through 0.22 μm sterilized syringe filters (BS-PES-22, Labgic Co., Ltd., Beijing, China) and added to the KC-H medium to create Se concentrations of 0, 5, 10, 20, 40, 60, 80, 100, and 160 mg/L. *Chlorella vulgaris* K-01, initially at ~0.1 g/L dry weight (DW). The Se treatment experiment was extended for ten days, and the strain was maintained in a rotary shaker incubator without light (26 °C, 130 rpm) (ZCZY-CN, Shanghai ZHICHU Instrument Co., Ltd., Shanghai, China).

### 2.2. Measurement of Microalgal Biomass

Samples were collected daily and filtered through a suction filter (GM-0.33A, Tianjin Jinteng Experiment Equipment Co., Ltd., Tianjin, China) with 0.45 μm membrane filters (pre-weighted), and freeze-dried to determine DW. Biomass data for the Se treatment groups at 192 h were extracted, and nonlinear curve fitting was performed to obtain the EC50 value.

### 2.3. Growth Parameters Calculation

Growth parameters were calculated based on biomass data. Specific productivity (SP), global productivity (GP), and maximum productivity were calculated using the following equations:

SP = (x_2_ − x_1_)/(t_2_ − t_1_) (1)

GP = (x_f_ − x_0_)/(t_f_ − t_0_) (2)

x_1_ and x_2_ represent the biomass concentrations (g/L) at the assay times t_1_ and t_2_ (d), respectively. x_0_ refers to the initial biomass concentration (g/L), and x_f_ is the biomass concentration on the tenth day. Additionally, t_f_ and t_0_ are the times (d) corresponding to x_0_ and x_f_. To determine the maximum productivity (g/L/d), the GP equation was employed during the exponential phase [38].

### 2.4. Gaussian Process (GP) Regression Analysis

The growth curves of *Chlorella vulgaris* K-01 under different selenium (Se) concentrations were analyzed using Gaussian process (GP) regression, following procedures and algorithms from a prior study [39]. Comparative hypotheses testing was conducted between the 0 mg/L Se concentration and others. The null hypothesis (H0) posits that only the time variable explains the variation in biomass changes, whereas the alternative hypothesis (H1) postulates that both time and Se concentrations contribute to the variation in biomass. The log Bayes Factor threshold of 0.937 was applied, and results exceeding this threshold indicate that hypothesis H1 should be accepted, suggesting a statistically significant difference (*p* < 0.05).

### 2.5. Glucose Detection and Evaluation

Samples collected every 48 h were centrifuged (4 °C, 5000× *g* for 10 min), and glucose in the medium was detected by glucose detection kits (Applygen Co., Ltd., Beijing, China). Glucose consumption rate (Gr) and the glucose-to-algae conversion rate (GTAr) were calculated according to a previous study [31]. The formula was listed as follows:

Gr = ΔCglucose/Δt (mg/L/h)(3)

GTAr = ΔCalgae/ΔCglucose (g/g)(4)

ΔCalgae and ΔCglucose (mg/L) represent the changes in the concentrations of algae and glucose, respectively. Δt indicates the corresponding period (h).

### 2.6. SEM Microscopy

On the seventh day of cultivation, a three-microliter sample of the medium from the 60 mg/L Se treatment group was extracted for scanning electron microscopy (SEM). The sample underwent centrifugation at 5000× *g* for 5 min, and the supernatant liquid was discarded, leaving the algal cell pellet. The pellet was washed three times with PBS buffer (pH = 6.4). Subsequently, the algal cell pellet was re-suspended in 1 mL of 2.5% glutaraldehyde and incubated at 25 °C for 120 min to fix the cell morphology. The glutaraldehyde was removed by centrifugation at 5000× *g* for 5 min, followed by three washes with PBS buffer. Next, a series of ethanol concentrations (30%, 50%, 70%, 95%, and three times 100% *v*/*v*) were sequentially added, with each step involving a 10-min incubation to dehydrate the cells. Afterward, the samples were affixed to a sample stage using electrically conductive paste and sputter-coated with gold palladium using an ion sputtering instrument (BAL-TEC SCD005, Micro Surface Engineering, Inc., Los Angeles, CA, USA). Finally, the sample stage containing the samples was observed using a scanning electron microscope (Regulus8100, Hitachi, Japan).

### 2.7. Intracellular Selenium Detection through ICP-MS

The microalgal cell pellets were washed three times with PBS buffer to remove residual extracellular contents. Subsequently, the pellets were freeze-dried. To determine the total Se concentration in the biomass, ten micrograms of the dried biomass were digested with nitric acid and H_2_O_2_ (3:1 *v*/*v*) using a microwave digestion system (Multiwave 7000, Anton Paar, Ashland, VA, USA). The digestion procedure involved a gradual heating process for 25 min until reaching 230 °C, followed by a 20-min incubation at 230 °C and a gradual cooling period for 15 min. The digested samples were then transferred to a 15 mL centrifuge tube and diluted to 10 mL with 1% HNO_3_. For the determination of inorganic Se concentration in the biomass, ten micrograms of the dried biomass were dissolved in 10 mL of ultra-pure water and subjected to two cycles of high-pressure homogenization using a high-pressure cell disruptor (CF1 and CF2 Cell Disruptor, Constant Systems Ltd., Daventry, UK) at 5 °C and 30 kpsi. The samples were then centrifuged at 20 °C for 10 min at 3000 g to obtain the supernatant. Subsequently, 10 mL of cyclohexane (GC grade) was added and vortexed. After 10 min, the liquid was stratified, and the water phase was collected. This water phase was acidified with 15% hydrochloric acid and filtered through 0.22 μm Millipore filters (adapted from Sun et al. [40]). After the pre-treatment procedures, all samples were filtered using 0.22 μm syringe filters and analyzed using inductively coupled plasma-mass spectrometry (ICP-MS) (Thermo Scientific XSERIES, Thermo Fisher Scientific Inc., Carlsbad, CA, USA). The amount of organic Se was calculated by subtracting the inorganic Se from the total Se. The Se concentration in the medium (supernatant) was expressed as mg/L, while the concentration in the microalgal biomass was expressed as μg/g DW.

### 2.8. Extracellular Residual Selenium Determination through ICP-OES

At the end of the Se treatment experiment (240 h), samples were collected for each treatment and centrifuged at 4 °C, 5000× *g* for 10 min. The supernatant was filtered using 0.22 μm syringe filters and analyzed using an inductively coupled plasma optical emission spectrometer (ICP-OES) (Plasma 3000 ICP-OES, NCS Testing Technology Co., Ltd., Beijing, China).

### 2.9. Statistical Analysis

All treatments were conducted in triplicates. Mean ± standard errors (SEM) were calculated and illustrated in graphs. The data were analyzed by one-way analysis of variance (ANOVA) at a 95% confidence interval (a = 0.05). The statistical analysis and graph-making were conducted using the software package Origin 2023 (OriginLab Corporation, Northampton, MA, USA).

## 3. Results and Discussion

### 3.1. Se Dose–Response of Chlorella vulgaris K-01 under Heterotrophic Medium

**Figure 1:**
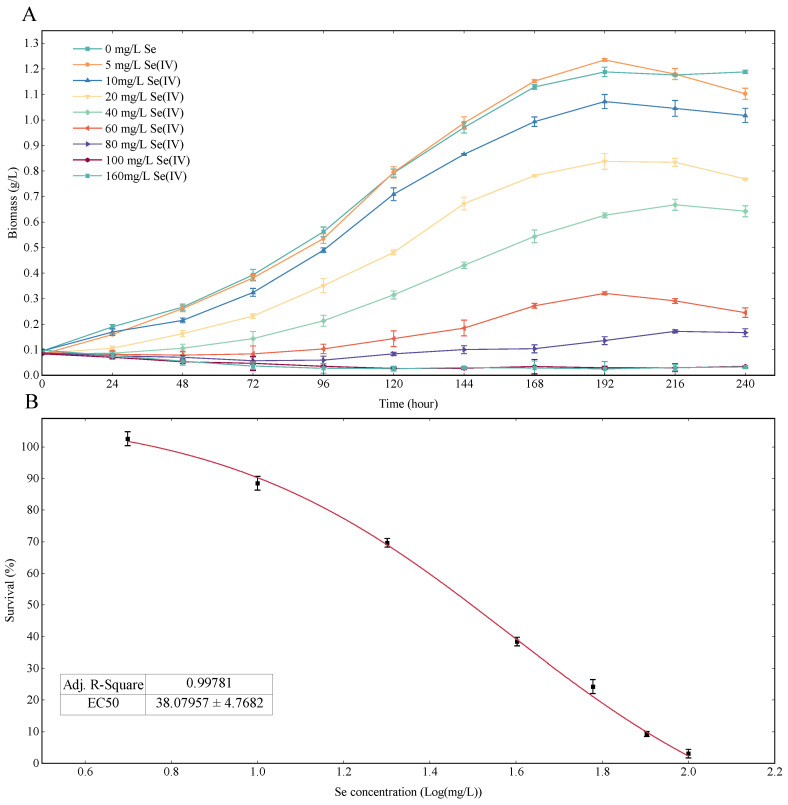
(**A**) Heterotrophic growth response of *Chlorella vulgaris* K-01 under different concentrations of selenium. (**B**) The survival rate of the algal cells under different concentrations of selenium at 192 h for the heterotrophic growth response. Nonlinear fitting of the data points is shown as a red line and the corresponding EC50 and Adj. The R-square value is shown in the table below the fitted curve.

Figure 1 illustrates the heterotrophic Se dose–response curve of *Chlorella vulgaris* K-01. In Figure 1A, the growth curve for the 5 mg/L Se treatment closely aligns with the control group. At 192 h, the 5 mg/L Se treatment group achieved the highest yield at 1.24 g/L, while the control group yielded 1.20 g/L. As Se concentration increased, the growth curve slopes gradually declined, and growth of *Chlorella vulgaris* K-01 completely stagnated when the Se concentration reached 100 mg/L. Figure 1B shows the non-linear curve fitting of biomass data at 192 h, revealing an EC50 value of 38.08 mg/L for *Chlorella vulgaris* K-01 under Se stress.

The EC50 value signifies the strain’s tolerance to Se stress, a crucial factor for assimilating organic Se in aqueous conditions. *Chlorella vulgaris* K-01 demonstrates superior Se tolerance compared to *Chlorella vulgaris* HNUFU001 (EC50 = 30 mg/L Se(IV)). Other studies, though lacking EC50 values, contribute valuable insights. For instance, Mylenko et al. [41] studied the Se tolerance of *Chlorella vulgaris* G120 in heterotrophic culture and found that the strain could resist 16 mg Se per 1 g of microalgal biomass. Pires et al. [38] studied the Se tolerance of *Chlorella vulgaris* 0007CA and found 10 mg/L Se could compromise the growth performance. Compared with previous reports, *Chlorella vulgaris* has better Se resistance in heterotrophic culture. For other microalgal species, EC50 for photoautotrophic Se have been frequently reported such as *Scenedesmus quadricauda* (EC50 = 3.9 mg/L Se (IV)) [42], *Haematococcus Pluvialis* (EC50 = 24 mg/L Se (IV)) [25], *Nannochloropsis oceanica* (EC50 = 12.94 mg/L Se(IV)) [43], *Chlorella pyrenoidosa* (EC50 = 5.37 mg/L Se(IV)) [44], and *Chlamydomonas reinhardtii* (2.9 mg/L Se (IV)) [45]. *Chlorella vulgaris* K-01 could be considered a Se-tolerant strain as the EC50 value for it was 1~14 fold compared with the above-mentioned species.

### 3.2. Growth Rate Analysis

**Figure 2:**
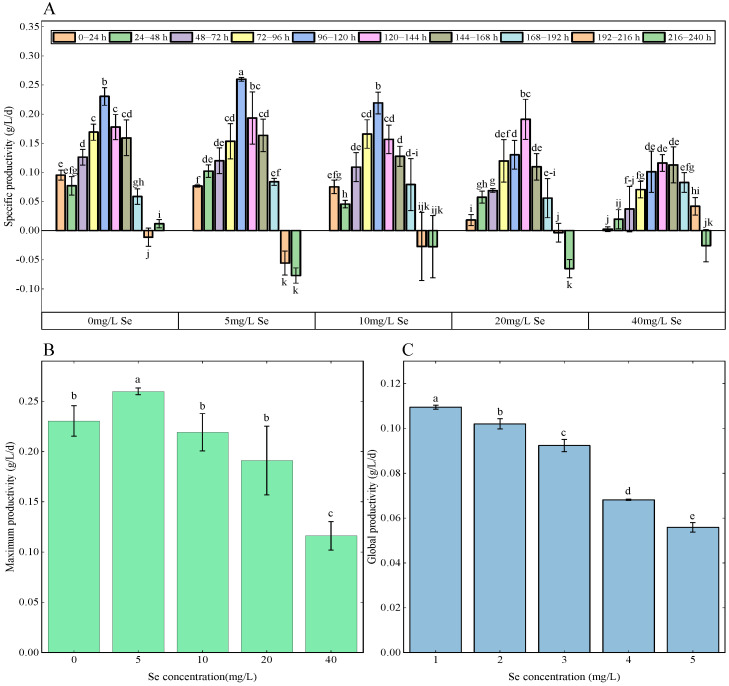
(**A**) Specific productivity; (**B**) maximum productivity; (**C**) global productivity of *Chlorella vulgaris* K-01 under the different concentrations of Se treatment in the heterotrophic regime. Differences in lower-case letters above the error bars indicate a significant difference (*p* < 0.05).

Figure 2 depicts the specific productivity, maximum productivity, and global productivity of *Chlorella vulgaris* K-01 under 0, 5, 10, 20, 40 mg/L Se treatment in the heterotrophic regime. In Figure 2A, specific productivities were calculated by 24 h intervals. Overall trends across groups were similar, with productivity starting low, gradually increasing, peaking, and then declining, potentially reaching negative values around 192–240 h. Initial productivity (0–24 h) significantly decreased with increasing Se concentration, ranging from 0.10 g/L/d for the control group to 0.01 g/L/d for the 40 mg/L Se treatment group (*p* < 0.05). Specific productivity for the control group significantly increased on 48–72 h intervals. For 5, 10, 20, and 40 Se treatment groups, the first significant increase in productivity to around 0.1 g/L/d was observed at 24–48 h, 48–72 h, 72–96 h, and 96–120 h, respectively. Figure 2B highlights maximum productivity with the 5 mg/L Se treatment group achieving significantly higher values (0.26 g/L/d) compared with other groups (*p* < 0.05). There was no significant difference in maximum productivity for the 0, 10, and 20 mg mg/L Se treatment groups, while the 40 mg/L Se treatment group exhibited a significant decrease (*p* < 0.05). Global productivity, illustrated in Figure 2C, for *Chlorella vulgaris* K-01 significantly decreased as the concentration of Se in the medium increased (*p* < 0.05).

The overall productivity trends align with the canonical heterotrophic microalgae growth pattern, encompassing lag, exponential, stationary, and death phases [46]. It seems that the Se treatment at the sub-lethal level did not affect the growth curve patterns of the species. However, higher Se concentrations (>5 mg/L) may impact the survival rate, causing a prolonged lag phase. This delay could result from the activation of detoxification mechanisms, allocating cellular resources to defense-related enzyme synthesis rather than biomass growth, as observed in previous reports. The prolonged lag phase could also be postulated by the activation of the detoxification mechanism that allocates more cellular resources to the cellular defense against stress rather than biomass growth. This phenomenon was also reported in previous studies [31,36,41]. Maximum productivity in the 5 mg/L Se treatment group was significantly more enhanced than other treatments with higher Se concentrations, indicating a low level of Se could be conducive to biomass production. Similarly, in the literature, Zhao et al. [44] reported a significant increase in the growth rate of *Chlorella pyrenoidosa* under 2 mg/L sodium selenite. Babaei et al. [47] found the synergistic effect of the administration of 16 mg Se/g DW on *Chlorella vulgaris* strain R117 (at 500 μmol photons/m^2^/s). This study suggested that low Se levels (5 mg/L) were able to enhance biomass production, consistent with previous findings for *Chlorella*.

### 3.3. Growth Curves Analysis Using GP Regression

**Figure 3:**
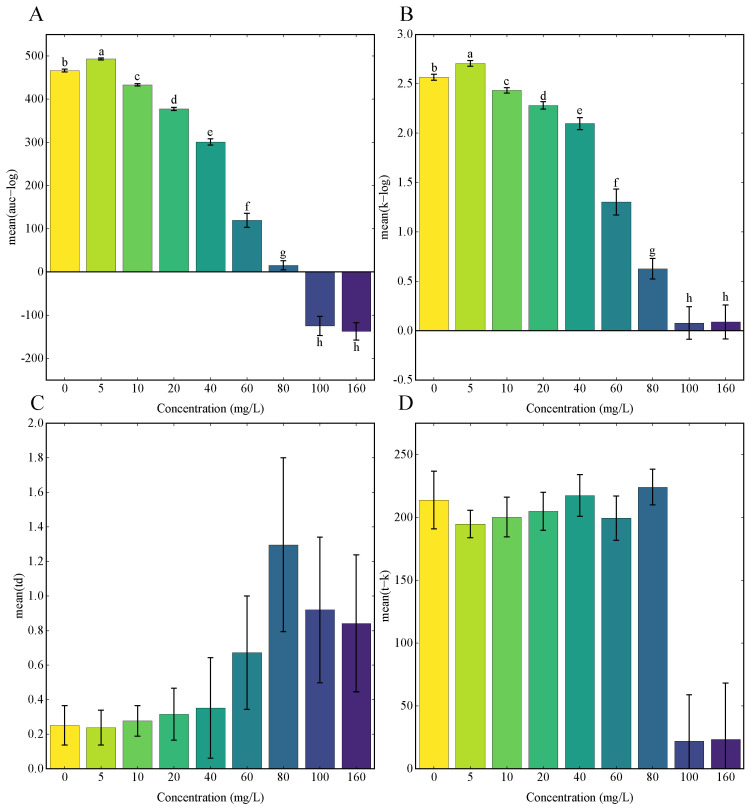
Growth parameters of the growth curve of *Chlorella vulgaris* K-01 under different concentrations of Se calculated with the Gaussian process regression model. (**A**) Area under the curve (in units of log biomass); (**B**) carrying capacity (in units of log biomass); (**C**) doubling time; (**D**) the time point at which carrying capacity is reached. Differences in lower-case letters above the error bars indicate a significant difference (*p* < 0.05).

GP regression, a recently developed method for microbial growth curves analysis, was applied to study the impact of stress on microbial population growth [39]. Although the model is accurate in the analysis of time-series data [48], GP regression on the analysis of microalgae responding to abiotic stresses has rarely been reported. This study implemented the GP regression on the analysis of growth curves of *Chlorella vulgaris* K-01 under Se stress in heterotrophic conditions (Figure 3).

As Se concentration increased, the area under the curve (Figure 3A) decreased and eventually became negative at 100 mg/L Se. Similarly, the carrying capacity (Figure 3B) decreased beyond 5 mg/L Se. Furthermore, the doubling time (td) (Figure 3C) increased, indicating reduced growth capacity. The time point of reaching carrying capacity (Figure 3D) decreased at 100 and 160 mg/L Se, signifying an early onset of the stationary phase. Hypothesis testing using GP regression log Bayes Factors demonstrated that Se significantly affects the microalgal growth. In comparison, the log Bayes Factors for Se treatment groups of 0–5, 0–10, 0–20, 0–40, 0–60, 0–80, 0–100, and 0–160 mg/L were 1.619, 23.385, 65.13, 81.359, 98.247, 115.035, 115.175, and 112.9, respectively. It is obvious that the log Bayes Factor for all Se treatment groups exceeded the threshold value of 0.937, indicating the Se treatment significantly affects the growth capacity of *Chlorella vulgaris* K-01 under a heterotrophic regime (*p* < 0.05).

The results from GP regression revealed that a low level of Se treatment could increase the biomass accumulation (area under the curve) and the carrying capacity of *Chlorella vulgaris* K-01 in heterotrophic culture. Notably, lower Se levels enhanced biomass accumulation and carrying capacity, suggesting potential applications in Se enrichment fermentation [31].

### 3.4. Microalgal Morphology Disruption under Se Stress

**Figure 4:**
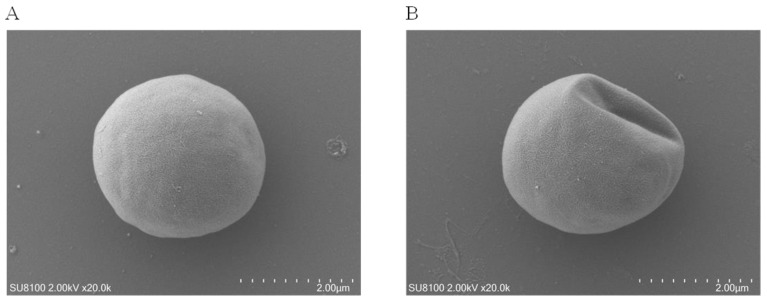
Scanning electron microscope image showing the morphology of *Chlorella vulgaris* K-01. (**A**) The normal coccoid shape in the control group; (**B**) The irregular algal cell surface under 60 mg/L selenium treatment in the heterotrophic medium.

Scanning electron microscopy (SEM) was employed to characterize the morphology of *Chlorella vulgaris* K-01 under Se stress, as shown in Figure 4. It indicated that Se stress led to a distortion of the typical coccoid shape of *Chlorella* (Figure 4A), resulting in a shrunken and irregular algal cell surface landscape (Figure 4B). These findings contribute to a more comprehensive understanding of microalgal responses to abiotic stress, as disruptions in cell morphology have implications for physiological states [31,36]. The SEM images of *Chlorella vulgaris* K-01 in response to Se under heterotrophic conditions filled an information gap in the literature, emphasizing the impact of Se stress on microalgal morphology.

### 3.5. Glucose Consumption Analysis

**Figure 5:**
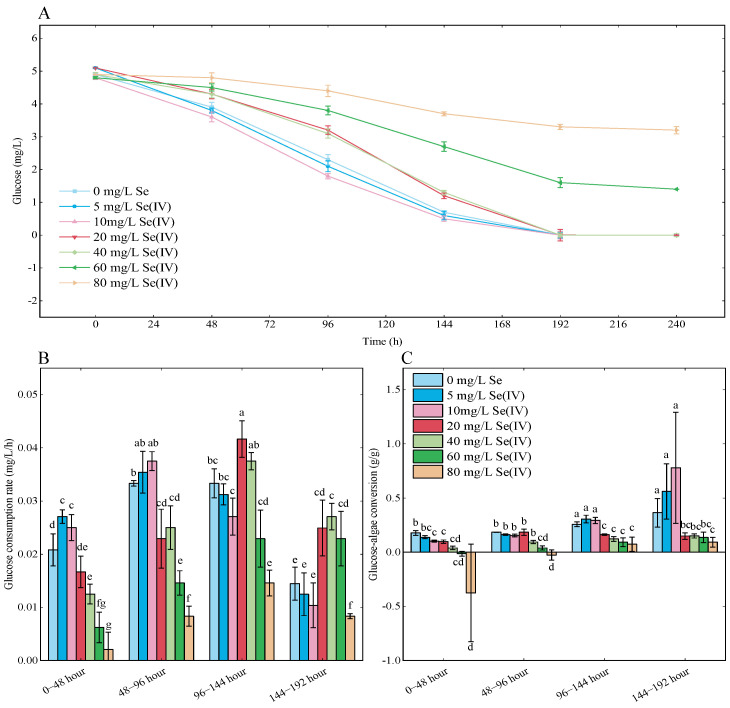
Glucose consumption and conversion of *Chlorella vulgaris* K-01 in heterotrophic medium under different concentrations of selenium. (**A**) Glucose concentration in the medium; (**B**) glucose consumption rate; (**C**) conversion of glucose into algal biomass. All data are presented as mean ± SEM. Differences in lower-case letters above the error bars indicate a significant difference (*p* < 0.05).

Figure 5A shows the glucose concentrations in the medium for Se treatment groups. The curves for glucose consumption followed a similar trend with the residual glucose in the medium gradually decreasing as the cultivation time increased. Glucose in the medium was completely consumed at 192 h for 0, 5, 10, 20, and 40 mg/L Se treatment groups, which coincided with the arrival of the stationary phase in growth curves (Figure 1A). It was found that the glucose consumption in treatment groups with Se concentration above 60 mg/L was inhibited, with 3.30 mg/L remaining glucose for the 80 mg/L Se treatment group at the end of the experiment. Corresponding glucose consumption rate and glucose-algae conversion were calculated and visualized in Figure 5B and Figure 5C, respectively. On 0–48 h, the glucose consumption rate for the 0, 5, 10, 20, 40, 60, and 80 mg/L Se treatment group were 0.02, 0.03, 0.03, 0.02, 0.01, 0.006 and 0.002 mg/L/h, respectively. The glucose consumption rate significantly decreased in groups with Se concentrations above 40 mg/L (*p* < 0.05). Overall glucose consumption at 48–144 h (exponential phase) increased for each group and decreased at 144–192 h. The 0–48 h glucose-algae conversion were 0.18, 0.14, 0.10, 0.10, 0.04, −0.01, and −0.39 g/g for the 0, 5, 10, 20, 40, 60, and 80 mg/L Se treatment groups, respectively. The glucose-algae conversion for the 0, 5, 10 mg/L Se treatment groups was significantly increased to 0.27, 0.31, 0.30 g/g (*p* < 0.05) on 96–144 h.

In comparison to phototrophic growth, heterotrophic conditions considerably enhance growth rates, final cell number, and cell mass in microalgae cultures [49]. Glucose, proven to support the continuous growth of microalgae in darkness [50], is the most effective carbon source for *Chlorella* in heterotrophic conditions [51]. This study implemented glucose as the sole carbon source for heterotrophic cultivation of *Chlorella* vulgaris K-01. The strain effectively consumed glucose in the medium to support the production of microalgal biomass. When the Se concentration was below 40 mg/L, 5 mg/L glucose was depleted. In contrast, consumption of glucose became incomplete in the 60 and 80 mg/L Se treatment groups, indicating the inhibition of carbon metabolism when Se stress was elevated. Mylenko et al. [41] reported the effect of Se on *Chlorella vulgaris* G120 in a heterotrophic regime and found that 16 mg/g Se in the medium could inhibit glucose consumption [52,53]. Other studies on the heterotrophic growth of *Chlorella* spp. also observed depletion of the glucose in the medium, the concurrence of glucose depletion, and the advent of the stationary growth phase on the growth curves [54]. The decrease in glucose consumption rate and glucose-algae conversion could be considered further evidence of Se stress, suggesting that Se could affect the carbon metabolism of microalgae in heterotrophic conditions. The possible connection of Se and carbon metabolism has been reported on other species such as *Oryza sativa* and *Gallus gallus* [55,56] and could be a promising research perspective for microalgae.

### 3.6. Se Incorporation and Removal

**Figure 6:**
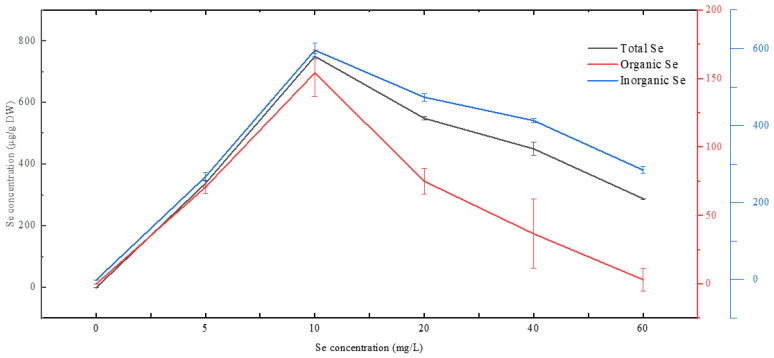
Concentrations of different forms of selenium in *Chlorella vulgaris* K-01 dried biomass after 240 h of cultivation in the heterotrophic regime.

Figure 6 depicts the intracellular Se incorporation of *Chlorella vulgaris* K-01 after 240 h of heterotrophic culture. Intracellular total Se, organic Se, and inorganic Se increased with rising Se concentration in the medium, reaching their peaks at the 10 mg/L Se treatment group. The maximum values for total Se, organic Se, and inorganic Se were 749.61, 154.00, and 595.60 μg/g DW, respectively.

**Figure 7:**
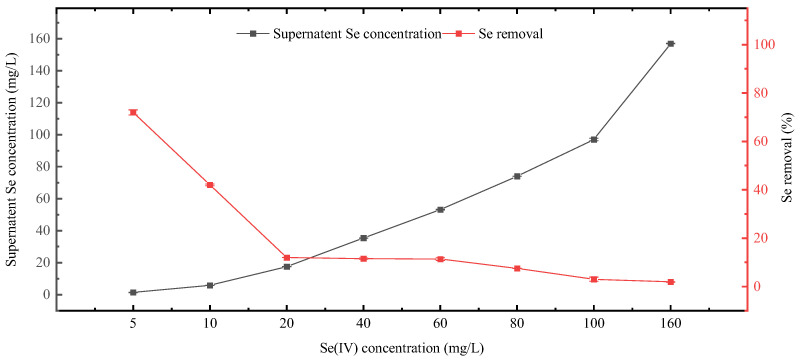
Supernatant Se concentration and Se removal rates under different concentrations of Se treatment.

Se removal capacity of *Chlorella vulgaris* K-01 was evaluated by measuring the residual Se concentration in the medium, and the Se removal rate was subsequently calculated (Figure 7). Supernatant Se concentration for the 5, 10, 20, 40, 60, 80, 100, and 160 mg/L Se treatment groups were 1.40, 5.80, 17.60, 35.40, 53.20, 74, 97, and 156.10 mg/L, respectively. Correspondingly, the removal rates were 72%,42%, 12%, 11.5%, 11.33%, 7.50%, 3%, and 1.9%, respectively. *Chlorella vulgaris* K-01 shows higher Se removal capacity at Se concentration below 10 mg/L, and the Se removal rate dropped to around 10% when Se concentration reached 20 mg/L.

Se incorporation into microalgal cells involves multiple pathways, substituting sulfur through sulfur assimilation pathways [57]. Recent discoveries of Se-specific pathways highlight the complexity of Se incorporation [58,59,60]. Different microalgal species have varied capacities of Se incorporation [61]. *Chlorella vulgaris* K-01 incorporate considerable amounts of organic Se compared to *Chlorella vulgaris* HNUFU001 (108.84 μg organic Se/gDW) [31], *Chlorella pyrenoidosa* (72 ± 0.1 μg organic Se/gDW) [44], and *Chlorella vulgaris* 0007CA (86.37 ± 0.060 μg organic Se/gDW) [38]. *Chlorella vulgaris* K-01 demonstrates a noteworthy capacity for Se incorporation, particularly in organic Se, surpassing other microalgal species

The Se removal capacity of *Chlorella vulgaris* K-01 was assessed by measuring residual Se concentration in the medium, and the corresponding removal rates were calculated (Figure 7). For Se treatment groups below 10 mg/L, *Chlorella vulgaris* K-01 exhibited higher Se removal rates, with a noticeable drop to around 10% as Se concentration reached 20 mg/L.

In comparison to previous studies, *Chlorella vulgaris* K-01 demonstrated a slightly elevated Se removal capacity in heterotrophic conditions. The Se removal rates reported for *Chlorella vulgaris* HNUFU001 [31] ranged from 5 to 40% when treated with 1–100 mg/L Se, whereas *Chlorella vulgaris* K-01 displayed a slightly higher level of Se removal capacity under similar conditions. The Se removal efficiency is crucial for evaluating the potential of microalgae in bioremediation applications. *Chlorella vulgaris* K-01 showcases robust Se incorporation, especially in organic Se, and exhibits effective Se removal capacity in heterotrophic conditions. These findings underscore the strain’s potential applications in both Se bioaccumulation and bioremediation processes.

## 4. Conclusions

This study provides novel insights into the impact of selenium (Se) on *Chlorella vulgaris* K-01 in a heterotrophic condition. Utilizing Gaussian process regression analysis on growth curves, we observed that low level Se treatment promotes biomass accumulation and enhances the carrying capacity of *Chlorella vulgaris* K-01 in heterotrophic culture. This emphasizes the strain’s potential for robust growth under controlled Se conditions. Furthermore, an in-depth analysis of productivity and residual glucose demonstrated the strain’s ability to tolerate low concentrations of Se without compromising its energy and carbon metabolism. The findings suggest that *Chlorella vulgaris* K-01 maintains its metabolic functionality even under mild Se stress, making it a resilient candidate for diverse cultivation conditions. By employing ICP-MS and ICP-OES, we determined that the strain displays a robust capacity for Se enrichment. *Chlorella vulgaris* K-01 can incorporate up to 154 μg/g DW of organic Se, showcasing its potential as a valuable source for Se-containing nutraceuticals. Additionally, the strain demonstrated an impressive 72% Se removal rate, highlighting its potential in bioremediation applications. These findings not only contribute to our understanding of *Chlorella vulgaris* K-01′s response to Se stress but also provide a valuable reference for the development of heterotrophic microalgal production techniques for Se-containing nutraceuticals. Future research should focus on determining the forms of organic selenium in the biomass of *Chlorella vulgaris* K-01, as well as on developing bioactive selenium-containing natural products, seleno-polypeptides, and selenium-containing polysaccharides.

## Data Availability

Data is contained within the article.
